# Autoregressive and Residual Index Convolution Model for Point Cloud Geometry Compression

**DOI:** 10.3390/s26041287

**Published:** 2026-02-16

**Authors:** Gerald Baulig, Jiun-In Guo

**Affiliations:** 1 College of Electrical & Computer Engineering, National Yang Ming Chiao Tung University, No. 1001, University Road, East District, Hsinchu City 300, Taiwan; gerald.baulig.ee07@nycu.edu.tw; 2 Institute of Electronics Engineering, National Yang Ming Chiao Tung University, No. 1001, University Road, East District, Hsinchu City 300, Taiwan; 3 eNeural Technologies Inc., 2 F., No. 19-1, Chuangxin 1st Rd., Baoshan Township, Hsinchu City 300092, Taiwan

**Keywords:** data compression, point cloud, LiDAR, autonomous driving, robotic, virtual reality

## Abstract

This study introduces a hybrid point cloud compression method that transfers from octree-nodes to voxel occupancy estimation to find its lower-bound bitrate by using a Binary Arithmetic Range Coder. In previous attempts, we demonstrated that our entropy compression model based on index convolution achieves promising performance while maintaining low complexity. However, our previous model lacks an autoregressive approach, which is apparently indispensable to compete with the current state-of-the-art of compression performance. Therefore, we adapt an autoregressive grouping method that iteratively populates, explores, and estimates the occupancy of 1-bit voxel candidates in a more discrete fashion. Furthermore, we refactored our backbone architecture by adding a distiller layer on each convolution, forcing every hidden feature to contribute to the final output. Our proposed model extracts local features using lightweight 1D convolution applied in varied ordering and analyzes causal relationships by optimizing the cross-entropy. This approach efficiently replaces the voxel convolution techniques and attention models used in previous works, providing significant improvements in both time and memory consumption. The effectiveness of our model is demonstrated on three datasets, where it outperforms recent deep learning-based compression models in this field.

## 1. Introduction

Point clouds have become a fundamental representation for three-dimensional data in a wide range of applications, including autonomous driving, robotics, remote sensing, and virtual and augmented reality [1,2,3]. Their ability to capture fine-grained geometric structures makes them indispensable wherever accurate spatial understanding is required. At the same time, raw point clouds are typically large in size and irregular in structure, resulting in substantial storage, transmission, and processing costs. This challenge has made Point Cloud Geometry Compression (PCGC) a critical research topic, with the goal of reducing data volume while preserving geometric fidelity.

Most PCGC pipelines follow a common structure consisting of spatial discretization, structure modeling, probability estimation, and entropy coding. First, continuous 3D point coordinates are quantized and discretized into a structured domain, most commonly using voxels organized in regular grids or hierarchical octrees.

Voxels serve as the volumetric analog of pixels, representing discrete cubic cells that partition three-dimensional space. This discretization transforms an unordered and irregular point set into a finite, countable symbol space with explicit spatial relationships, enabling statistical modeling and compression. For geometric representation, each voxel can be encoded using a single binary variable v∈[0,1], which we refer to as voxel occupancy. Subsequently, voxels are modeled to exploit spatial redundancy across neighboring cells and across resolutions. Finally, a context model estimates symbol probabilities, which are compressed using entropy coding techniques such as arithmetic coding.

Among discretization schemes, octrees are particularly well suited for point cloud geometry compression. An octree is a hierarchical spatial data structure that recursively subdivides space into eight equally sized cubic regions. At each node, the occupancy state of its eight children is encoded as an 8-bit symbol o∈[1,…,255], which we denote as an occupancy symbol. Starting from a root node that spans the entire bounding volume, nodes are either marked as occupied or subdivided depending on the presence of geometric content. Dense regions are represented at finer resolution, while empty space is encoded compactly. This adaptive representation allows octrees to efficiently capture geometric structure while enabling hierarchical context modeling and scalable decoding across resolutions, making them a standard backbone for many geometry codecs.

Traditional PCGC methods, such as Google’s Draco [4] and MPEG’s G-PCC [5], rely on handcrafted context models defined over tree or graph-based spatial structures. These models encode designed heuristics about spatial dependencies, resulting in stable performance and predictable decoding behavior. However, their probability estimation is fundamentally limited by fixed rules and manually selected context, restricting their ability to adapt to diverse geometric distributions. As a result, a trade-off emerges: G-PCC achieves strong Rate–Distortion (R–D) performance at the cost of high decoding latency, while Draco prioritizes fast decoding with reduced compression efficiency.

Recent advances in deep learning have significantly improved PCGC by replacing handcrafted context models with data-driven probability estimation. Voxel-based approaches [6,7,8,9] employ 3D convolutions or masked autoregressive models to predict voxel occupancy on regular grids, while octree-based methods [10,11,12,13] learn hierarchical context models aligned with tree structures. Hybrid techniques [14,15] combine voxel and octree representations to leverage the strengths of both. Despite their effectiveness, most neural approaches face a fundamental trade-off between compression performance, decoding latency, and memory consumption. In particular, models with strong spatial modeling capabilities often rely on expensive 3D convolutions, attention mechanisms, or fully autoregressive decoding, which substantially increase inference time.

In earlier work [16], we introduced multi-index convolution as a lightweight entropy model for PCGC. The key idea was to approximate multi-dimensional spatial context using multiple deterministic 1D index orderings, enabling efficient context aggregation without resorting to costly 3D operators. While this approach demonstrated favorable complexity characteristics, its compression performance lagged behind state-of-the-art methods. We identify three primary causes:

1. The absence of coherent autoregressive feedback among sibling nodes;
2. An insufficiently expressive backbone architecture;
3. The inherently more difficult objective of predicting octree-node occupancy symbols rather than voxel occupancy.

In this work, we present MIC-OPCCv2, a substantially improved multi-index convolution framework that addresses these limitations and narrows the gap between compression efficiency and decoding latency. By reformulating geometry coding as a voxel occupancy prediction problem, we enable the use of an efficient progressive grouping strategy [9], which introduces autoregressive dependencies while preserving partial parallelism during decoding. This strategy allows early decisions to benefit from refined context while accelerating later decoding stages. Furthermore, we propose a novel blender & distiller network architecture, which strengthens residual learning inspired by ResNet [17] and adopts a hierarchical feature aggregation mechanism reminiscent of U-Net [18]. Unlike conventional encoder–decoder designs, this architecture employs a voting-based probability aggregation scheme that explicitly integrates contributions from multiple network depths, improving robustness to large entropy variations across the sparse and dense regions of a point cloud. Finally, we extend multi-index convolution by incorporating the breadth-first traversal order alongside axial index permutations, enhancing diagonal spatial context capture without incurring the computational overhead of sparse 3D convolution or attention.

The main contributions of this paper are summarized as follows:

1. Extended Multi-Index Convolution: An enhanced index-based convolution framework that expands spatial receptive fields through multiple traversal orders, including breadth-first traversal, while maintaining low computational complexity.
2. Blender & Distiller Architecture: A hierarchical residual aggregation and voting mechanism that stabilizes probability estimation across varying entropy regimes.
3. Progressive Grouping Strategy: An autoregressive yet partially parallel decoding scheme that balances contextual refinement and decoding efficiency.

Together, these components enable MIC-OPCCv2 to achieve competitive or state-of-the-art R–D performance across both sparse LiDAR point clouds and dense object reconstructions, while significantly reducing decoding complexity compared to transformer- and sparse convolution-based models. Experimental results further demonstrate that the proposed method generalizes well across heterogeneous data distributions and offers a favorable trade-off between compression efficiency and runtime.

The remainder of this article is organized as follows. Section 2 reviews related work in deep learning-based point cloud compression. Section 3 introduces the theoretical background and entropy modeling framework. Section 4 details the proposed MIC-OPCCv2 architecture. Section 5 describes the experimental setup and evaluation protocol. Finally, Section 6 presents the results, followed by discussion and conclusions.

## 2. Related Work

In PCGC, we distinguish between *sparse* and *dense* point clouds primarily reflecting differences in spatial distribution and structural completeness.

Sparse point clouds—typically acquired by mobile LiDAR systems [19,20,21,22]—contain relatively few points per unit volume, exhibiting large empty regions, anisotropic sampling patterns, and strong non-uniformity caused by occlusions and long-range sensing. They generally represent expansive, concave outdoor environments in which surfaces are only partially and unevenly observed.

In contrast, dense point clouds—such as voxelized human-body scans or multi-view reconstructions [23,24]—provide near-contiguous sampling of predominantly convex shapes, forming compact, high-density clusters with rich geometric detail and stable local neighborhoods.

Both types pose greater challenges for contextual modeling, occupancy prediction, and entropy coding due to their irregular structure and large proportion of data. This compels most methods to transform them into more uniform representations. These approaches estimate occupancy probabilities through context modeling and achieve compression via entropy coding. Typically, context models rely on spatial feature analysis, such as via 3D voxel convolutions or handcrafted features that incorporate occupancy indicators and quantization levels. More recent studies have shifted toward autoregressive techniques, which enhance compression efficiency but substantially increase inference time. While fully autoregressive models can be parallelized during encoding, decoding must proceed sequentially for each point $v_{t}$, since it depends on the prior state $v_{<t}$. This sequential dependency has motivated semi-autoregressive approaches that use grouping strategies to enable partial parallel decoding with minimal trade-offs.

Depending on their representation strategies, methods can be broadly classified into four categories: *tree-based*, *voxel-based*, *projection-based*, and *point-based* approaches. Tree-based models represent geometry via KD-trees [25] or octrees [26] and predict node occupancy hierarchically. Voxel-based methods [6] operate on quantized voxel grids with 3D convolutional networks. Projection-based approaches [27,28] compress 2D depth maps, while point-based models encode learned point-wise features directly.

### 2.1. Traditional Methods

**Figure 1:**
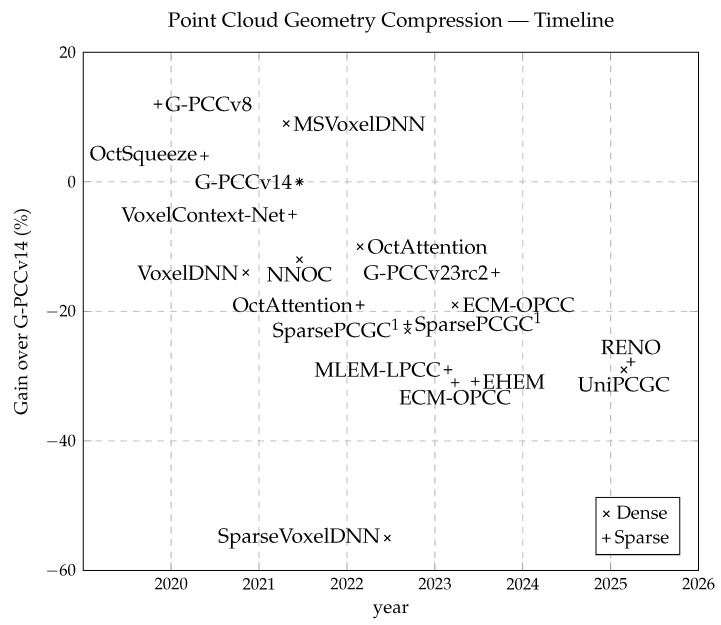
The timeline of key publications in lossless point cloud geometric compression over the past half decade, illustrating the steady performance improvements achieved relative to the G-PCCv14 baseline. ^1^ SparsePCGC was originally published in November 2021 with comparatively lower performance due to hardware limitations at the time. With the advent of newer GPUs such as the NVIDIA RTX 4090, larger model configurations and reduced inference times have become feasible, resulting in significantly improved compression performance.

Traditional methods for PCGC, such as Google’s Draco [4] and MPEG’s G-PCC [5], are widely adopted due to their interpretability, stability, and general effectiveness. Both convert point clouds into KD-trees and use node occupancy for context modeling. Projection-based methods, such as V-PCC [28], project 3D data into 2D representations for conventional image compression, but they suffer from projection artifacts and partial information loss. All these codecs rely on heuristically designed contexts limited to small neighborhoods, restricting overall performance. This has prompted a shift toward learned entropy models that automatically infer context information using deep neural networks. The timeline in Figure 1 highlights this steady performance improvement of deep learning-based models over traditional G-PCCv14 [5], for both sparse and dense point clouds.

### 2.2. Voxel-Based Entropy Models

These methods employ 3D convolutions over voxelized occupancy grids. However, due to the cubic complexity $O(n^{3})$, high-resolution voxelization can quickly exceed hardware capacity. Nguyen et al. introduced VoxelDNN [6], inspired by PixelCNN [29], which partitions point clouds into 64^3^ voxel blocks and uses masked 3D convolutions in a fully autoregressive manner to predict voxel occupancy probabilities. Kaya et al. proposed NNOC [30], which explores the voxel grid layer by layer, but both models remain fully autoregressive and hence computationally expensive. MSVoxelDNN [7] partially relaxes the autoregressive dependency, achieving parallelism at the cost of higher bitrates.

Sparse convolution frameworks such as the Minkowski Engine [31,32] allow high-resolution processing. This enabled Wang et al. to develop SparsePCGC [8], capable of 12-bit precision using sparse convolution with an 8-stage autoregressive model. Later, UniPCGC [9] proposed a refined grouping strategy, arguing that early-stage voxels provide underrepresented spatial information. By skipping select voxels in initial groups and expanding later stages, UniPCGC improved both compression ratio and decoding time compared to SparsePCGC. SparseVoxelDNN [33] is namely the transition of VoxelDNN [6] from dense convolution to sparse convolution.

### 2.3. Octree-Based Entropy Models

Octree-based approaches decompose point clouds hierarchically, enabling localized feature extraction per node. OctSqueeze [10] was the first end-to-end octree-based model, predicting the 8-bit occupancy symbol of each node using a Multi-Layer Perceptron (MLP) that processes ancestor features. However, its lack of neighborhood search and autoregression limited effectiveness, and later voxel-based approaches surpassed it. Recent octree models, including OctAttention [11], ECM-OPCC [12], and EHEM [13], leverage attention mechanisms such as transformers to capture hierarchical dependencies. They convert octrees into deterministic sequences (e.g., breadth-first order), allowing transformers to model autoregressive dependencies efficiently. Yet, the breadth-first ordering only approximates neighborhood relations and does not always reflect true spatial proximity. While these transformer-based methods excel in sparse point clouds, they are computationally heavy. For example, ECM-OPCC [12] achieves strong compression performance but requires 19.5 s for decoding and nearly exhausts 40 GB of GPU memory, limiting real-time applicability. EHEM [13] introduces a *hierarchical attention mechanism* designed to accelerate decoding by efficiently expanding the receptive field. It first merges the features of sibling nodes before applying the transformer, and then up-samples them back to their original resolution. This hierarchical fusion strategy effectively broadens the spatial context at low computational cost, demonstrating particular efficiency for sparse point clouds.

### 2.4. Hybrid Entropy Models

Hybrid models combine multiple representations—such as octrees, voxelization, and spatial coordinates—offering a balance between granularity and efficiency. VoxelContextNet [14] encodes octrees but converts each node into small voxel blocks $\left(11^{3}\right)$ for 3D convolutional feature extraction. The more recent RENO [15] uses sparse convolution with embedded occupancy features (32 channels per voxel) and two autoregressive stages. Our proposed MIC-OPCCv2 consumes point-based features while maintaining an octree structure, but encodes each occupancy symbol bit-by-bit in a voxel-wise manner.

### 2.5. Summary

In summary, Table 1 provides a structured comparison of leading deep learning-based PCGC methods. We categorize these models according to four key aspects:

1. Type of input features;
2. Main context modeling component;
3. Type of probability output;
4. Level of autoregression.

The final two columns report their compression performance on sparse versus dense point clouds and their decoding times. All results are cited faithfully from original sources. It is evident that fully autoregressive models achieve strong compression but at impractical decoding times. Semi-autoregressive methods address this limitation through architectural enhancements or model scaling. Finally, voxel-based approaches tend to perform better on dense point clouds, whereas octree-based methods excel on sparse data.

Projection-based methods are excluded from this survey due to their inherent limitations. For instance, RIDDLE [27] applies only to single range image frames from individual sensors, whereas many real-world point clouds are composites from multiple scans and sensors [20,21,23,24].

Despite significant progress, existing methods continue to face key trade-offs between compression efficiency, decoding speed, and memory consumption. Fully autoregressive models achieve superior bitrates but suffer from prohibitively long decoding times, while semi-autoregressive or sparse convolution methods often sacrifice accuracy for faster inference. Transformer-based octree models, though highly expressive, require extensive computational resources and large memory footprints, making them unsuitable for real-time applications or deployment on embedded systems. To address these challenges, we propose MIC-OPCCv2, a lightweight and computationally efficient hybrid entropy model based on multi-index 1D convolution. Our approach captures spatial context through dynamically ordered convolutions, achieving fast decoding with competitive compression performance—bridging the gap between traditional octree-based and voxel-based neural codecs.

## 3. Background

Our method builds on the same foundational principles as previous works, leveraging Shannon’s theorem [34], which defines the entropy *h* of a symbol *s* as

(1) $$h\left(s\right)=-\log_{2}p(s).$$

This formulation defines the entropy *h* as the theoretically required number of bits to represent a symbol *s*, where the probability p(s) lies within the range 0<p(s)<1. During decoding, however, the true probability distribution p(s) is uncertain and can only be approximated by a stochastic, heuristic, or empirical model. We express this approximation as

(2) $$q\left(x\right)=\hat{y},$$

where y is a one-hot encoded vector representing the ground truth symbol *s*, and $\hat{y}$ is the estimated probability distribution inferred from the contextual input x. Our goal is to optimize $q_{\theta }\left(x\right)$—a parameterized empirical model, such as one driven by deep learning—by minimizing the cross-entropy between y and $Q_{\theta }\left(x_{i}\right)$:

(3) $$H\left(y,x\right)=-\sum_{i=1}^{n}y_{i}\log Q_{\theta }\left(x_{i}\right),$$

where H(y,x) measures the divergence between the true and estimated distributions. An Arithmetic Encoder [35] can then transform the symbol *s* into a compact bitstream, with the efficiency of the encoding directly tied to the accuracy of the probability estimate $Q_{\theta }\left(x_{i}\right)={\hat{y}}_{i}$.

### 3.1. Binary Arithmetic Coding

Arithmetic coding is a form of entropy encoding used in lossless data compression. Unlike Huffman coding [36], which assigns discrete bit patterns to each symbol, arithmetic coding encodes an entire message into a single fractional number within the interval [0,1). This approach allows for fractional bit efficiency, resulting in compression rates close to the theoretical entropy limit.

In arithmetic coding, a message is represented by a progressively refined numerical interval. Each new symbol subdivides the current interval into smaller partitions according to its estimated probability. The more probable a symbol is, the smaller the range reduction it induces—thus requiring fewer bits to encode. This recursive subdivision continues for all symbols in the message, producing an increasingly precise numerical representation. When the final interval is specified to sufficient precision, its binary representation serves as the compressed output.

Binary Arithmetic Coder (BAC), a simplified form of arithmetic coding, performs recursive interval subdivision based on binary decisions between the most probable symbol and the least probable symbol. The interval width corresponds to the product of the symbol probabilities, and the binary representation of the final interval asymptotically approaches the Shannon theorem’s lower bound on entropy. A key advantage of BAC is its clean separation between modeling and coding, allowing a probability model to be optimized independently from the coding engine—a property central to our proposed approach.

### 3.2. Fast Octree Coder

**Figure 2:**
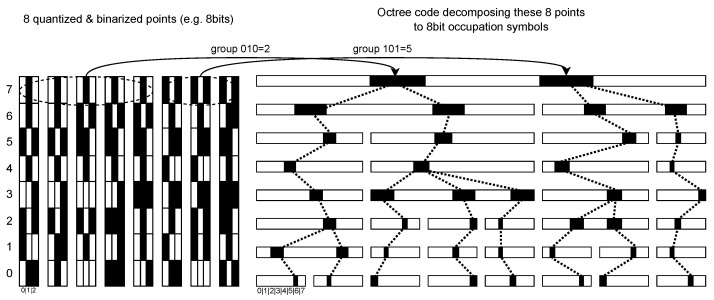
Fast Octree Coding. Any octree level at depth *t* can be constructed instantly with a complexity of $O(\log_{8}n)$ by grouping points according to their 3t most significant bits, enabling efficient hierarchical partitioning of the quantized point cloud.

An octree provides an efficient means of organizing unstructured and unsorted data through recursive spatial partitioning [26]. However, costly spatial computation and floating-point arithmetic can be avoided by Fast Octree Coding (FOC) as shown in Figure 2. FOC simply groups a pre-quantized point cloud *Q* based on the *t* most significant bits of each component in a quantized point $q=p_{b_{L-t}}$, where *t* reflects the current quantization level and tree depth. Each group raises a bit on the 8-bit occupancy symbol *s* according to its octant signature at position *t*. This algorithm allows fast random access to any depth-level in the tree at a complexity of $O(\log_{2}n)$

A breadth-first traversal of the octree, expressed as $S=\{s_{0}=\left(s_{0},\ldots ,s_{j}\right),\ldots ,s_{L}\}$, typically yields much lower entropy per symbol *s* than the raw spatial coordinates p=(x,y,z) of a point cloud *P*.

Earlier octree-based models, such as OctSqueeze [10], included the real-valued positions of each octree-node as features. However, the absence of real position values in recent works [9,11,12,13,15] implies that this feature is less effective for spatial context modeling. Instead, embedded occupancy flags, 3-bit octants, and quantization depth provide more discriminative and compact features.

## 4. Methodology

**Figure 3:**
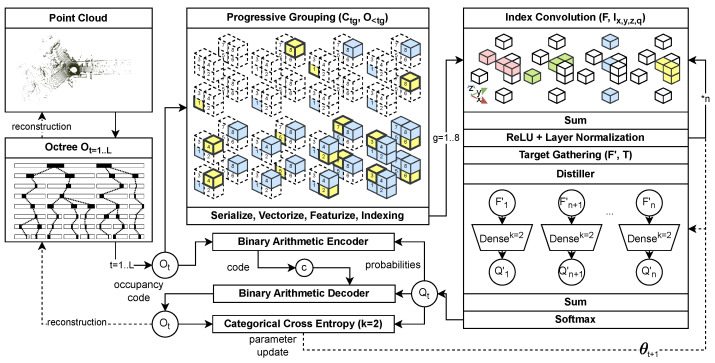
Overview of the MIC-OPCCv2 architecture. The input point cloud is quantized and binarized to form an octree representation. Each octree-node expands into eight voxel candidates, which are arranged into eight autoregressive groups following the progressive grouping scheme. Each group is processed by the blender & distiller network using multi-index convolution to estimate occupancy probabilities. These probabilities are consumed by a BAC during compression and are supervised through categorical cross-entropy during training. The resulting occupancy symbols can be converted back into an octree and subsequently into a reconstructed quantized point cloud. Arrows indicate data flow, unless further descriped. All core modules are further descriped in Section 4.

Building upon the limitations identified in prior research, we propose MIC-OPCCv2—an enhanced version of our lightweight multi-index convolutional entropy model [16] designed for efficient octree-based point cloud compression. An overview of the framework is provided in Figure 3. The architecture integrates four key components:

1. A *progressive grouping* scheme, which introduces autoregressive dependencies between voxel candidates at identical resolution levels;
2. A *blender & distiller* network, which refines hierarchical representations through residual feature aggregation;
3. A *multi-index convolution* backbone, enabling spatially coherent context extraction at minimal computational cost;
4. A *binary location* encoder, serving as a compact and expressive geometric feature representation.

Together, these components allow MIC-OPCCv2 to achieve competitive or state-of-the-art compression performance while operating with significantly lower memory usage and substantially reduced decoding complexity compared to existing neural PCGC methods.

The following sections detail the core elements of the proposed codec. Section 4.1 introduces the multi-index convolution mechanism. Section 4.2 and Section 4.3 describes the blender & distiller network and the progressive grouping strategy. Section 4.4 outlines the construction of binarized location features, and Section 4.5 presents the training objective and loss formulation.

### 4.1. Index Convolution

**Figure 4:**
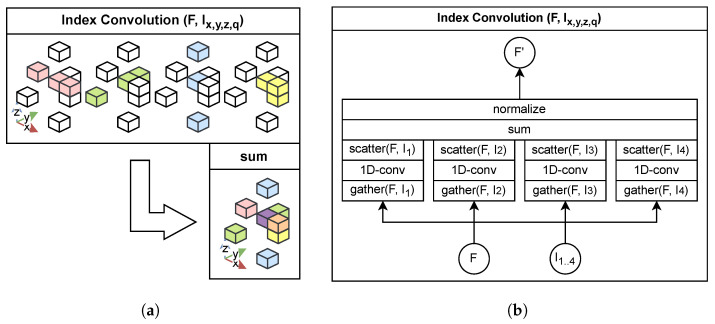
Illustration of the proposed multi-index 1D convolution framework, which reorders spatial features along multiple dimensions to form directionally variant receptive fields. Each convolution output is reverted and aggregated in the original order to accumulate contextual information in all directions. (**a**) presents a visual example of the multi-index convolution using four different orderings (x,y,z,q), highlighted in distinct colors and combined by summation. (**b**) provides a schematic overview of the processing pipeline, with arrows indicating the data flow.

Transformer-based methods [11,12,13] process octree-nodes sequentially following a breadth-first traversal order. However, this neighborhood ordering is not always spatially consistent, as nearby points or voxels may appear far apart in the traversal sequence as octree-nodes. To overcome this limitation, we introduce a multi-index 1D convolution operation, illustrated in Figure 4.

For each spatial dimension, an arg-sort operation generates an index that defines the processing order. This index is created once at the beginning. The point features are then reordered according to this index before applying 1D convolution. After convolution, the outputs are reverted to their original order using the inverse index and aggregated. Repeating this process across all dimensions progressively enlarges the model’s receptive field, enabling the aggregation of broader spatial dependencies.

Our indexed convolution can be interpreted as an approximated low-complex *k*-nearest-neighbor lookup along fixed axes, effectively merging adjacent voxels in sorted order. Unlike previous methods that rely on zero-padding for empty voxels within a fixed receptive window, our approach ensures that only existing voxels contribute to the receptive field. This dynamic adaptation allows the model to handle sparse regions more effectively by implicitly adjusting the receptive field size.

### 4.2. Blender & Distiller

Samples in PCGC exhibit a wide range of entropy characteristics. Low-resolution samples tend to emit low entropy, while high-resolution samples approach near-random distributions with substantially higher entropy. Consequently, prior research has emphasized the value of residual network architectures to accommodate such variability [9,17,33]. The underlying intuition is that low-entropy samples can be effectively modeled with shallow networks, whereas high-entropy samples require deeper networks capable of capturing more complex causal dependencies.

**Figure 5:**
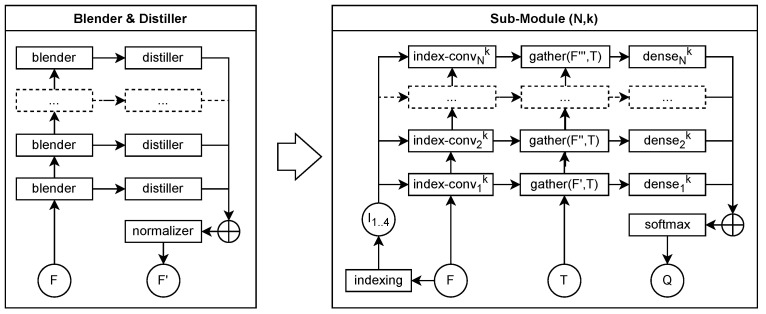
The proposed *blender & distiller* architecture is a strongly residual framework inspired by U-Net [18], but differs by incorporating a multi-stage voting mechanism on the output side, where each distiller contributes to the final probability estimation. The left panel presents an abstract schematic of the architecture, while the right panel illustrates a concrete implementation example. Dashed boxes denote placeholders for repeated layers, and arrows indicate the direction of data flow.

Voxel occupancy estimation, as formulated in PCGC, is conceptually analogous to image segmentation. In this domain, U-Net architectures [18,37] have demonstrated superior performance compared to conventional ResNet-based models, primarily due to their ability to combine multi-scale contextual information through skip connections. Inspired by these findings, we propose a blender & distiller architecture, illustrated in Figure 5.

In our design, the blender layers act as recursive feature aggregators that combine information across different receptive fields. Aggregation can be achieved through summation, concatenation, convolution, or attention-based fusion. In our case, multi-index convolution serves as the primary aggregation mechanism, progressively refining spatial context representations across hierarchical depth. Each blender layer thus produces increasingly complex hidden features, with deeper layers contributing to broader spatial understanding.

The distiller layers, on the other hand, are responsible for extracting probability estimations from each blender stage. Their outputs are subsequently combined through a normalized summation, effectively forming a collective “vote” across all hierarchical levels of the network. This mechanism enforces contribution from every stage, ensuring that intermediate representations meaningfully participate in the final prediction. Empirically, this voting-based mechanism proves highly effective for PCGC, as it maintains strong contextual awareness across both local and global spatial scales.

Unlike the U-Net [18], our design omits connections between distiller inputs, simplifying the data flow while maintaining hierarchical feature consistency. For binary classification tasks, each distiller outputs two positive unconstrained logits. This formulation allows a distiller to either abstain from contributing (by emitting near-zero magnitudes) or to dominate the final consensus when confident. Through backpropagation, each distiller dynamically adjusts its contribution to the aggregate prediction, facilitating adaptive behavior across samples of varying entropy.

Formally, the aggregated output of all *N* distillers is expressed as

(4) $$\begin{array}{c} F_{t,g}^{N+1}=\sum_{i=1}^{N}{\mathrm{Distiller}}_{i}\left(F_{t,g}^{i}\right), \end{array}$$

(5) $$\begin{array}{c} Q_{\theta }\left(C_{t,g}|O_{<(t,g)}\right)=\mathrm{SoftMax}\left(F_{t,g}^{N+1}\right), \end{array}$$

where ${\mathrm{Distiller}}_{i}\left(F_{t,g}^{i}\right)$ denotes the *i*-th distiller output operating on hidden features $F_{t,g}^{i}$ derived from the preceding blender layer. The softmax normalization converts the summed logits into a probability distribution $Q_{\theta }\left(C_{t,g}|O_{<(t,g)}\right)$, which represents the estimated occupancy likelihood for voxel candidates $C_{t,g}$ conditioned on the previously decoded occupancies $O_{<(t,g)}$.

These probabilities directly feed into the categorical cross-entropy loss described in Section 4.5, ensuring that learning is guided by the aggregated multi-scale consensus of all distillers. In this way, the *blender & distiller* network forms a mathematically consistent bridge between spatial feature extraction and probabilistic entropy modeling, enabling efficient and robust learning across diverse octree resolutions.

### 4.3. Progressive Grouping

To incorporate autoregressive dependencies while avoiding the high latency of fully sequential decoding, we adopt a grouped decoding strategy inspired by UniPCGC [9], which itself extends the SparsePCGC [8] framework. Rather than decoding voxels strictly one-by-one, grouping strategies partition voxel candidates into subsets that are decoded iteratively, enabling a controlled trade-off between contextual refinement and parallelism. We collectively refer to these approaches as variants of autoregressive grouping strategies.

**Figure 6:**
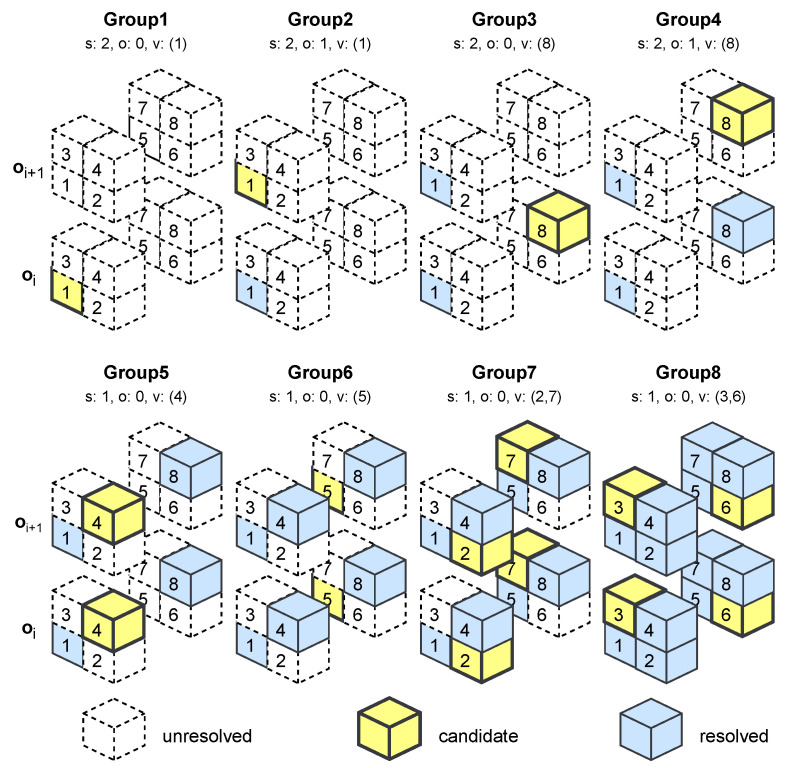
Progressive grouping strategy of MIC-OPCCv2. Each octree-node $o_{i}$ expands into a block of eight voxel candidates *v*. For every autoregressive group, voxel candidates are selected according to a stride *s*, an offset *o*, and a target index *v*. Early groups operate under limited spatial context and therefore decode only a subset of voxels, enabling more reliable probability estimation for later groups. As contextual information accumulates, later groups increase throughput by decoding multiple voxels concurrently.

In this work, we implement a progressive grouping strategy, illustrated in Figure 6, that dynamically adjusts the grouping granularity across decoding stages. The key motivation is that early decoding decisions are made under limited contextual information and therefore benefit most from fine-grained autoregressive conditioning, whereas later decisions can be decoded more aggressively once the occupancy distribution becomes increasingly constrained. Accordingly, the first decoding stages process a reduced candidate set by activating only every second voxel, which limits parallelism but maximizes contextual reliability. As decoding progresses, the grouping size is gradually increased, allowing more voxel candidates to be decoded per iteration while leveraging the richer context accumulated from earlier stages.

This progressive schedule differs from sequential grouping or fully autoregressive methods in that it explicitly adapts the balance between context accuracy and decoding throughput over time. It also avoids excessive sequential dependency chains, which would otherwise dominate decoding latency. Empirically, we observe that this ordering provides a favorable compromise between compression efficiency and runtime compared to alternative grouping strategies, such as sequential grouping [8] or fully voxel-wise autoregression [33]. Nevertheless, we note that the effectiveness of progressive grouping depends on the spatial distribution of occupied voxels: for highly dense or highly regular structures, overly aggressive grouping in later stages may reduce contextual precision.

Overall, the proposed progressive grouping strategy enables efficient autoregressive modeling with linear-time decoding complexity, while preserving most of the rate-distortion benefits of fine-grained context modeling.

### 4.4. Binary Location Encoding

**Figure 7:**
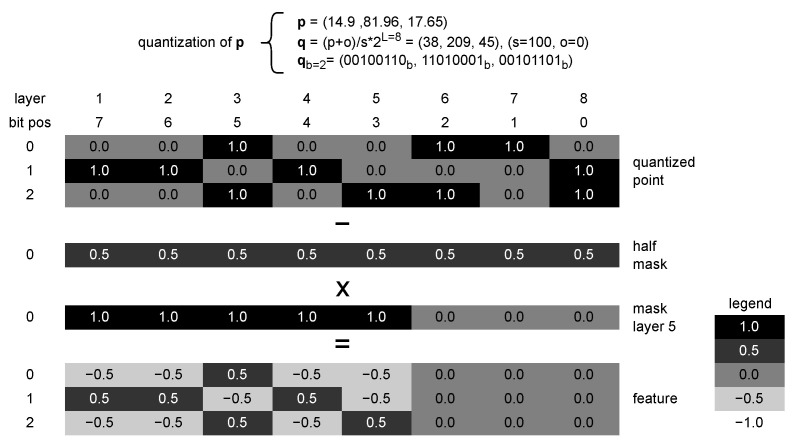
Illustration of the binarization, normalization, and masking process for feature generation at quantization level L=8 and current coding depth t=5. Bits beyond the current depth are masked to prevent access to unavailable information during decoding.

The preprocessing pipeline used for our model input is illustrated in Figure 7. Each point p=(x,y,z)∈R is first normalized and quantized according to

(6) $$\begin{array}{c} q=\left\lfloor \frac{2^{L}}{s}\left(p-o\right)\right\rfloor , \end{array}$$

ensuring all coordinates are positive and confined to the range $\left[0,2^{L}\right)$. A binarization function generates bit-level representations:

(7) $$\begin{array}{c} b_{i}\left(p\right)=\left\lfloor \frac{p}{2^{i}}\right\rfloor \;\mathrm{mod}\;2, \end{array}$$

and concatenation across axes produces hierarchical binary descriptors:

(8) $$\begin{array}{c} b_{L}\left(q\right)=\left[b_{L-1}\left(z\right),b_{L-1}\left(y\right),b_{L-1}\left(x\right),\ldots ,b_{0}\left(z\right),b_{0}\left(y\right),b_{0}\left(x\right)\right]. \end{array}$$

A mask $m_{td}$ ensures that only information up to the current decoding depth *t* is visible:

(9) $$\begin{array}{c} m_{i}=\left\{\begin{array}{cc} 1, & \mathrm{if}\;i<td,\\ 0, & \mathrm{otherwise} \end{array}\right.. \end{array}$$

Finally, the masked binary features are centered to [−0.5,0.5] to distinguish true zeros from masked bits:

(10) $$\begin{array}{c} F^{0}=\left\{\left(b_{L}\left(p_{j}\right)-0.5\right)m_{td}|\forall j\right\}. \end{array}$$

Using this binarization scheme, variant indices *I* can be derived for different voxel orderings, enabling diverse spatial perspectives in the multi-index convolution process. By summing the bits across the quantization levels *L* and dimensions *d*, we obtain an index $I_{q}$ corresponding to the breadth-first traversal order of an octree:

(11) $$\begin{array}{c} I_{q}=\left\{\sum_{k=0}^{L-1}\sum_{d=1}^{3}b_{t}\left(p_{d}\right)2^{3t+d-1}|\forall p=\left(x,y,z\right)\right\}. \end{array}$$

Alternatively, by summing the binary bits along dimensions first and then over quantization levels, we derive axial voxel orderings, such as $I_{x}$, $I_{y}$, and $I_{z}$:

(12) $$\begin{array}{c} I_{x}=\left\{\sum_{d=1}^{3}\sum_{t=1}^{L}b_{L-t}\left(p_{d}\right)2^{Ld-t}|\forall p=\left(x,y,z\right)\right\}, \end{array}$$

(13) $$\begin{array}{c} I_{y}=\left\{\sum_{d=1}^{3}\sum_{t=1}^{L}b_{L-t}\left(p_{d}\right)2^{Ld-t}|\forall p=\left(y,z,x\right)\right\}, \end{array}$$

(14) $$\begin{array}{c} I_{z}=\left\{\sum_{d=1}^{3}\sum_{t=1}^{L}b_{L-t}\left(p_{d}\right)2^{Ld-t}|\forall p=\left(z,x,y\right)\right\}. \end{array}$$

By cyclically swapping the dimensional components in p, we obtain different axial orderings for each dimension *d*. These variant indices serve as sorting references for our multi-index convolutions, allowing the model to perceive spatial relationships from multiple directional perspectives.

### 4.5. Loss Function

The training objective of MIC-OPCCv2 is to minimize the categorical cross-entropy loss between the predicted probability distribution $P_{t,g}$ and the true occupancy label $O_{t,g}$ for each voxel, across all resolution levels *t*, and autoregressive groups *g*. The loss function is formulated as

(15) $$\begin{array}{c} L_{t,g}=E_{O_{t,g}∼P_{t,g}}\left[-\log Q_{\theta }\left(C_{t,g}|O_{<(t,g)}\right)\right], \end{array}$$

where $Q_{\theta }$ represents our probability estimation model parameterized by θ, applied over the current voxel candidates $C_{t,g}$ and the already processed occupancy symbols $O_{<(t,g)}$ from previous decoding steps.

For each target voxel, the model outputs two class probabilities—occupied versus empty—using a softmax activation applied to the final feature representation $F_{t,g}^{N+1}$, as defined in Equation (5).

Each distiller layer ${\mathrm{Distiller}}_{i}$ implements a fully connected (dense) layer with kernel size k=2, using *SoftPlus* for unbound positive activation:

(16) $$\begin{array}{c} {\mathrm{Distiller}}_{i}\left(F_{t,g}^{i}\right)=\mathrm{SoftPlus}({\mathrm{Dense}}_{i}^{k=2}\left(F_{t,g}^{i}\right)). \end{array}$$

The input features $F_{t,g}^{i}$ correspond to the subset of encoded voxels $T_{t,g}$ at the current stage *i*, defined as

(17) $$\begin{array}{c} F_{t,g}^{i}=\left\{x|\forall n\in T_{t,g}:x_{n}\in F^{i}\right\}. \end{array}$$

The distillers extract two scalar outputs per voxel, corresponding to the probability logits of occupancy and emptiness. These logits are derived from the recursively accumulated hidden features $F^{i}$, obtained via the *blender layers*:

(18) $$\begin{array}{c} F^{i}={\mathrm{Blender}}_{i}\left(F^{i-1}\right), \end{array}$$

where each blender performs feature aggregation across all spatial indices using layer normalization and ReLU activation:

(19) $$\begin{array}{c} {\mathrm{Blender}}_{i}\left(F^{i-1}\right)=\mathrm{LayerNorm}\left(\sum_{d=1}^{4}\mathrm{ReLU}\left({\mathrm{IndexConv}}_{i,d}^{k=64}\left(F^{i-1},I_{d}\right)\right)\right). \end{array}$$

This formulation summarizes four index convolutions with different sorting orders $I_{d}$, starting from the initial feature set $F^{0}$:

(20) $$\begin{array}{c} F^{0}=\left\{b_{L}\left(p\right)|\forall p\in ⋃\left\{C_{t,g},O_{<(t,g)}\right\}\right\}, \end{array}$$

where $b_{L}\left(p\right)$ represents the binarized and serialized feature vector of spatial locations p. Here, $O_{<(t,g)}$ refers to all decoded voxels from prior steps, and $C_{t,g}$ represents candidate voxels generated for the current group. Our overall probability mass function is therefore

(21) $$\begin{array}{c} P_{\theta }\left(O\right)=\prod_{t=1}^{L}\prod_{g=1}^{G=8}Q_{\theta }\left(C_{t,g}|O_{<(t,g)}\right). \end{array}$$

This autoregressive structure allows MIC-OPCCv2 to iteratively refine spatial and contextual representations across layers and groups, improving the accuracy of occupancy probability estimation for each voxel candidate.

### 4.6. Model Splitting

**Figure 8:**
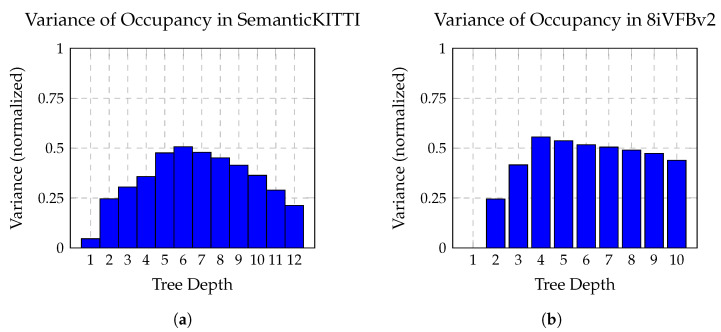
Variance in occupancy distributions across octree levels. (**a**) SemanticKITTI (sparse): entropy is low at shallow and deep levels, but peaks at mid-levels where structural variation is highest. (**b**) 8iVFBv2 (dense): occupancy distributions maintain consistently high variance starting from level 4 due to dense and surface-complete geometry.

Each octree level exhibits distinct entropy characteristics due to the hierarchical refinement of spatial resolution, as illustrated in Figure 8. The upper levels near the root generally show low entropy, since most nodes are fully occupied when the point cloud is centered. Intermediate levels contain the widest variety of occupancy patterns and therefore present the highest entropy. At deeper levels, although spatial precision increases, occupancy becomes sparse and the entropy decreases again, as fully occupied patterns are rare.

In principle, each octree level could be processed by an individually parameterized sub-module with dedicated convolutional and dense layers, enabling optimal adaptation to its entropy profile. However, such a design is computationally expensive, memory-intensive, and reduces parameter sharing, which may impair generalization. To balance expressiveness and efficiency, we group neighboring octree levels into a limited number of sub-modules, each tailored to a characteristic entropy regime, as follows:

- Module A (Shallow Levels): Processes the coarsest levels, where occupancy is dense and entropy is low. A compact configuration with fewer parameters is sufficient, as occupancy patterns are highly predictable.
- Module B (Intermediate Levels): Covers the layers with the greatest variability in occupancy and the highest entropy. Here, a more expressive representation is required. Since spatial structure is still relatively coarse, increasing the channel dimension *k* is more effective than increasing the number of layers *N*.
- Module C (Deep Levels): Handles the finest levels, where occupancy is sparse and spatial geometry must be inferred from broader context. In this regime, a large receptive field is essential, favoring a higher depth *N* of convolutional layers to propagate long-range dependencies.

This level-wise grouping strategy preserves predictive accuracy across the entire octree depth while controlling computational cost, enabling efficient modeling of both coarse global structure and fine-grained geometric detail. Moreover, the modular decomposition allows individual sub-modules to be fine-tuned, replaced, reused, or extended independently, facilitating adaptation of the codec to different application requirements such as memory constraints, target resolution, or operational latency.

## 5. Experiments

This section outlines the experimental setup used to evaluate the proposed MIC-OPCCv2 framework, including datasets, implementation details, and evaluation metrics. We then compare our method against representative state-of-the-art PCGC approaches on both sparse and dense benchmarks.

### 5.1. Datasets

To verify the robustness of MIC-OPCCv2 across different geometric structures, we evaluate the model on both sparse and dense point cloud datasets. For fair comparison, we adopt the same training and test splits as previous studies [12].

#### 5.1.1. SemanticKITTI

The SemanticKITTI dataset [19] consists of large-scale outdoor LiDAR scans captured from a rotating 64-beam sensor at 10 Hz. Each frame contains roughly $10^{5}$ points with coordinates p=(x,y,z,l), where the intensity *l* is not considered in our experiments. The point clouds are stored in 32-bit floating-point precision with a spatial resolution of $10^{-4}$ m and span up to approximately 270 m in diameter, requiring 18 bits per coordinate for lossless quantization. Following the standard protocol, sequences 00–10 are used for training, while the remaining sequences serve for testing.

#### 5.1.2. MPEG’s 8i Voxelized Full Bodies (8iVFBv2)

The 8iVFBv2 dataset [23] is used to evaluate performance on dense point clouds composed of voxelized human scans. Although the dataset contains both geometry and color, only geometry is considered in this work. The dataset is widely adopted due to its standardized quantization levels (10–12 bits), enabling controlled R–D comparisons. Following common practice [38], we evaluate four benchmark sequences: *Redandblack* and *Loot* (300 frames each), as well as single frames from *Thaidancer* and *Boxer*, while *Longdress* and *Soldier* are used for training.

### 5.2. Implementation Details

**Figure 9:**
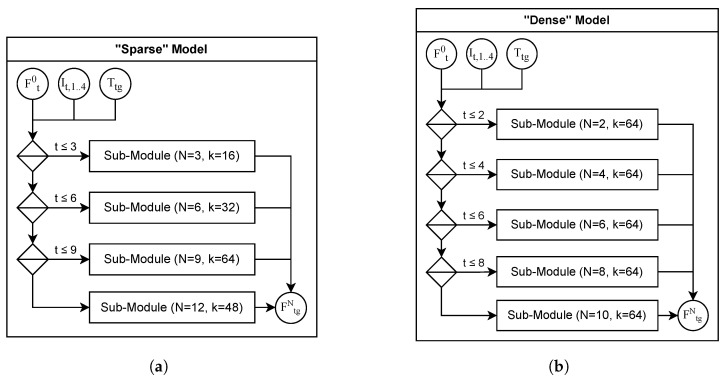
Model configurations for sparse and dense point clouds. (**a**) The sparse configuration employs four sub-modules, each with a distinct number of blender layers *N* and kernel size *k*, reflecting the varying entropy across octree depths in sparse point clouds. (**b**) The dense configuration assumes a more uniform occupancy distribution, using five sub-modules with fixed kernel size and gradually increasing depth to accommodate consistently high spatial detail.

MIC-OPCCv2 is implemented in TensorFlow 2.9 by using Python 3.6 and an integrated arithmetic coder TensorFlow Compression 2.9 provided by Ballé et al. [39]. We configure two model variants (as illustrated in Figure 9): one optimized for sparse point clouds and one for dense point clouds.

The *sparse model* supports a quantization precision of up to L=12 and is divided into four sub-modules (Section 4.6), with each sub-module responsible for three octree levels. These sub-modules use N=[3,6,9,12] blender layers and kernel sizes k=[16,32,64,48], respectively. The total number of trainable parameters remains below one million, and the memory footprint during decoding does not exceed 2 KB per point.

The *dense model* supports a quantization precision of up to L=10 and is composed of five sub-modules, each covering two octree levels. Here, the sub-modules use N=[2,4,6,8,10] blender layers with a constant kernel size of k=64. This configuration results in approximately 1.4 M trainable parameters, and the memory footprint during decoding remains below 4 KB per point.

### 5.3. Training Details

Both model variants are trained on single NVIDIA RTX 3090 GPU with 24 GB VRAM for approximately 30 epochs using the ADAM [40] optimizer, an initial learning rate of $10^{-4}$ with exponential decay (0.9), and a dropout rate of 0.01 to mitigate overfitting. The loss function follows Equation (15), computed over all octree levels and autoregressive groups. The final training accuracies reach about 88% (sparse) and 94% (dense) for occupancy prediction.

### 5.4. Baseline

We compare MIC-OPCCv2 against representative traditional and neural point cloud geometry compression methods, including G-PCCv14 [5], VoxelContextNet [14], SparseVoxelDNN [33], SparsePCGCv2 [8], UniPCGC [9], RENO [15], EHEM [13], and ECM-OPCC [12]. Methods that are either superseded by more recent variants or consistently underperform traditional codecs are omitted. Furthermore, approaches such as MuSCLE [41], RIDDLE [27], and MLEM-LPCC [42], which rely on latent feature reconstruction and therefore operate under a different compression paradigm, are excluded as well to maintain fair comparison.

### 5.5. Evaluation Metrics

We follow MPEG standard evaluation procedures [38] and report the following:

- Bits per point (bpp): average coding cost per point.
- D1-PSNR (dB): point-to-point PSNR for geometric reconstruction fidelity.
- D2-PSNR (dB): point-to-plane PSNR for surface reconstruction fidelity.
- BD-Rate (%): Bjøntegaard delta bitrate relative to a reference model [43].
- Runtime (s/frame): wall-clock encoding and decoding time.

For sparse compression on SemanticKITTI, we evaluate R–D using

(22) $$\begin{array}{c} \text{D1-PSNR}=10\log_{10}\frac{3p^{2}}{{\mathrm{MSE}}_{sym}}, \end{array}$$

(23) $$\begin{array}{c} {\mathrm{MSE}}_{sym}=\frac{1}{2}\left(\mathrm{MSE}\left(P,\hat{P}\right)+\mathrm{MSE}\left(\hat{P},P\right)\right), \end{array}$$

where

(24) $$\begin{array}{c} \mathrm{MSE}\left(P,\hat{P}\right)=\frac{1}{\left|P\right|}\sum_{i}\min_{j}{\left|p_{i}-{\hat{p}}_{j}\right|}^{2}, \end{array}$$

and the point-to-plane version uses

(25) $$\begin{array}{c} {\mathrm{MSE}}_{n}\left(P,\hat{P}\right)=\frac{1}{\left|P\right|}\sum_{i}\min_{j}{\left|n_{i}\left(p_{i}-{\hat{p}}_{j}\right)\right|}^{2} \end{array}$$

where $n_{i}$ is the normal-vector for $p_{i}$.

For lossless dense compression, we report BD-Rate relative to G-PCCv14.

## 6. Results

**Figure 10:**
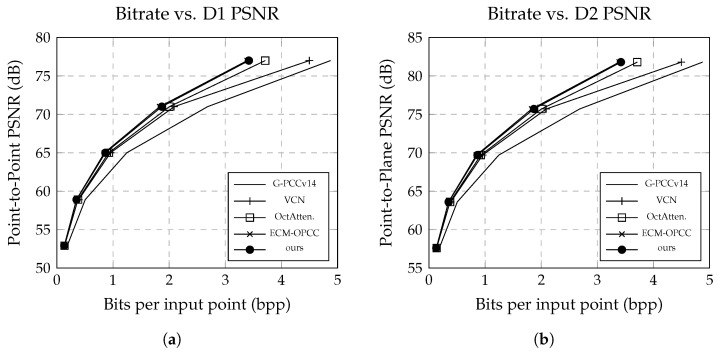
Results of our proposed MIC-OPCC model against state-of-the-art baselines on the SemanticKITTI dataset. While achieving comparable compression ratios to OctAttention, our model significantly outperforms it in both encoding and decoding speed. (**a**) shows the point-to-point distortion, and (**b**) the point-to-plane distortion.

Figure 10 illustrates the R–D of MIC-OPCCv2 on the SemanticKITTI dataset. With a quantization precision of L=12, our method achieves a bitrate of 3.41 bpp at a D2-PSNR of 82 dB, matching ECM-OPCC and thereby approaching the current state-of-the-art for sparse point cloud geometry compression.

VoxelContextNet, SparsePCGCv2, and ECM-OPCC all employ identical quantization strategies, allowing direct comparison of their PSNR values. In contrast, RENO and EHEM assume a peak signal of p=59.7 and apply different quantization configurations. To ensure a fair evaluation, we normalize their PSNR values to p=1 but adopt their quantization configurations accordingly, as reflected in Table 2. Despite not being trained at these distortion levels, MIC-OPCCv2 consistently outperforms both RENO and EHEM in R–D. This indicates that our model captures an underlying geometric representation that generalizes effectively across different resolutions, rather than relying on quantization-specific fitting.

Compared to MIC-OPCCv2, ECM-OPCC achieves superior compression performance on sparse point clouds mainly due to its attention-based context model, which can flexibly capture long-range and cross-level dependencies that are common in sparse geometries. Although ECM-OPCC relies on a linear octree traversal order that does not always preserve spatial neighborhood coherence, the global receptive field of self-attention compensates for this limitation by selectively emphasizing informative occupied regions. In contrast, MIC-OPCCv2 prioritizes decoding efficiency through structured index-based context modeling, which favors spatial coherence and linear complexity but may be less expressive in extremely sparse regimes. While ECM-OPCC could further benefit from more advanced indexing strategies, its transformer architecture incurs a significantly higher memory footprint and runtime cost, making MIC-OPCCv2 more suitable for large-scale or latency-constrained scenarios.

For dense geometry evaluated on the 8iVFBv2 dataset, MIC-OPCCv2 achieves an average bitrate of 0.50 bpp, outperforming UniPCGC and ECM-OPCC by approximately 1.14% and 11.2% in BD-Rate, respectively (see Table 3). SparseVoxelDNN attains superior compression performance on this dataset, which can be attributed to its fully voxel-wise autoregressive modeling that exploits dense and regular occupancy patterns more exhaustively. In contrast, MIC-OPCCv2 prioritizes decoding efficiency through grouped autoregression and linear complexity, which leads to a substantially reduced decoding time. Specifically, MIC-OPCCv2 decodes a frame in approximately 5 s, making it roughly ∼40× faster than SparseVoxelDNN and ∼4× faster than ECM-OPCC, while remaining slower than UniPCGC, which reports an average decoding time of 0.57 s per frame.

While the model achieves competitive or state-of-the-art R–D performance, the observed decoding latency highlights the need for further implementation-level optimization to fully exploit the theoretical efficiency of the architecture.

Overall, the results demonstrate that incorporating the progressive grouping strategy markedly enhances the effectiveness of multi-index convolution. MIC-OPCCv2 surpasses every baseline model in at least one of the two key performance dimensions: bitrate efficiency or decoding speed. This indicates that the model generalizes well across both sparse and dense point cloud distributions, achieving competitive or state-of-the-art R–D. However, the current decoding latency suggests that additional engineering optimizations are still needed to fully realize the theoretical efficiency of the proposed architecture.

## 7. Discussion

The experimental results from Section 6 demonstrate that MIC-OPCCv2 effectively bridges the gap between sparse convolutional and transformer-based PCGC models. By eliminating the attention overhead while preserving long-range context through multi-index convolution, the method achieves a favorable trade-off between R–D and computational efficiency. Moreover, its grouping scheme enables flexible decoding parallelism, bringing it closer to real-world applications, but still struggles with real-time performance due to implementation issues.

We discuss the latency problem by analyzing the theoretical runtime complexity in the following Section 7.1. Furthermore, in Section 7.2, we demonstrate and illustrate how the perceptive field of multi-index convolution expands in sparse and dense point clouds differently, to discuss strength and weakness of our methods, that may leave room for future investigation and improvement. To quantify the contribution of each proposed component, we present an ablation study in Section 7.3, demonstrating how voxel-wise prediction, the blender & distiller network, and progressive grouping each incrementally improve compression efficiency.

### 7.1. Runtime Complexity

The key idea of our multi-index convolution is to achieve a large receptive field in three-dimensional space while maintaining low computational complexity. Table 4 summarizes the theoretical complexity of transformers, Minkowski convolution, and our proposed multi-index convolution, alongside their measured decoding times.

It is evident that transformer architectures exhibit the highest computational complexity, making them the least efficient in this comparison. For each data point *n*, a transformer requires the computation of the key, value, and query layers, contributing 3ck, as well as the attention matrix with complexity $kw^{2}$, where *w* denotes the window size, *c* the number of input channels, and *k* the kernel size (i.e., number of output channels).

The Minkowski convolution, as defined by Choy C. et al. [31], generalizes the standard convolution to sparse domains, with a core complexity of $ckw^{d=3}$ that scales with the number of query points *m*. To maintain equivalence with dense convolutional outputs, the query count *m* increases across stacked layers due to the propagation of kernel offsets around each point *n*:

(26) $$m=n\frac{\left(N-1\right)w^{d}}{\epsilon },$$

where *N* is the number of layers and ϵ is a correction factor accounting for overlapping receptive fields. This factor ϵ tends to be smaller for dense point clouds (due to more overlap) and larger for sparse point clouds.

**Figure 11:**
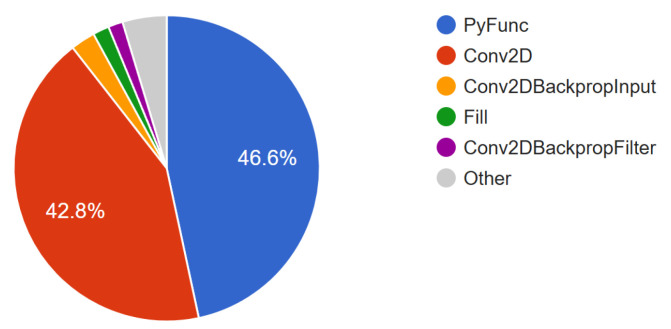
TensorBoard profiler pie chart illustrating the distribution of total on-device self-time by operation type (in percent). PyFunc denotes poorly supported Python operations—most notably gather and scatter routines—which account for a substantial portion of execution time and lead to idle periods on the acceleration hardware.

In contrast, the theoretical complexity of our multi-index convolution, given by n(ckwi), is substantially lower than that of Minkowski convolution, since $w\left(i=4\right)<w^{d=3}$, where *i* denotes the number of applied index orders *I* and *n* remains constant. However, despite this theoretical advantage, our current implementation exhibits a runtime roughly ten times slower than sparse Minkowski-based models. We attribute this discrepancy to non-optimized index reordering operations (see Figure 11), which cause extended GPU idle periods during the rearrangement of feature vectors *F* across index sets *I*. Ongoing optimization efforts aim to minimize these bottlenecks and further improve practical efficiency.

### 7.2. Perceptive Field

**Figure 12:**
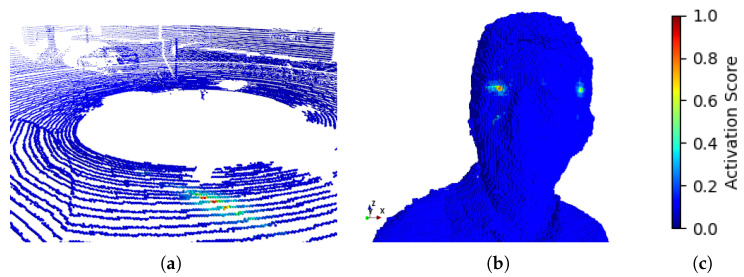
Theoretical activation score of the spatial receptive field generated by our multi-index convolution. (**a**) illustrates the receptive field on a sample from the SemanticKITTI dataset. (**b**) illustrates the receptive field on a sample from the MVUB dataset. (**c**) is the color scale used to illustrate the activation score.

Figure 12 illustrates the theoretical activation score of the spatial receptive field generated by our multi-index convolution, using a Gaussian filter. This visualization demonstrates how the proposed method effectively bridges large gaps in sparse point clouds, enabling better spatial context understanding in samples as in the SemanticKITTI dataset. On the other hand, this method occasionally extends beyond the object’s convex hull, unintentionally capturing points from the backside. Hence, the spatial compactness of this method is not guaranteed and intuitively highlights a limitation when applied to dense or closed-surface geometries as in samples from the MVUB [24] dataset. Nonetheless, we assume that the learned filters of our model should easily detect the un-similarity between these far distant voxels and lower their activation score.

### 7.3. Ablation Study

To better understand the contribution of each design choice in MIC-OPCCv2, we conduct a comprehensive ablation study that systematically evaluates the impact of the proposed architectural components and decoding strategies. In particular, we analyze how different autoregressive grouping schemes, depth-aware model splitting, and key module activations affect compression efficiency, estimation accuracy, memory consumption, and inference latency. Unless stated otherwise, all ablation experiments are performed under identical training settings to ensure fair and interpretable comparisons.

Table 5 evaluates the effect of different autoregressive grouping strategies on compression efficiency, estimation accuracy, memory consumption, and inference latency. Decoding without grouping achieves the lowest inference time but performs poorly in terms of bitrate and accuracy, indicating insufficient contextual modeling when all octree-nodes are decoded in a single stage. Sequential grouping substantially improves both bitrate and accuracy by enforcing strict voxel-wise autoregression per octree-node; however, this benefit comes at the cost of significantly increased inference time due to reduced parallelism. The proposed progressive grouping strategy provides the most favorable trade-off, achieving the lowest bpp and highest estimation accuracy while maintaining considerably lower inference latency than sequential grouping. Although progressive grouping introduces a moderate increase in peak memory usage compared to sequential decoding, it remains substantially more efficient than the no-grouping baseline, demonstrating that gradually expanding the decoding scope effectively balances contextual refinement and computational efficiency.

Table 6 investigates the impact of progressively splitting the network into depth-specific sub-modules along the octree hierarchy. Transitioning from a single monolithic model 0–12 to increasingly fine-grained depth splits consistently improves compression efficiency and estimation accuracy, reducing the bitrate from 4.169 to 3.715 bpp while increasing accuracy from 84.2% to 86.0%. At the same time, inference cost per point decreases monotonically, indicating that shallower, depth-specialized sub-modules enable more efficient computation. Notably, improvements saturate beyond the 0–4–8–12 configuration, where further splitting yields only marginal gains while maintaining similar memory requirements. This suggests that moderate depth-aware model splitting offers a favorable balance between compression performance, runtime efficiency, and memory consumption.

Table 7 disentangles the contributions of the major components introduced in MIC-OPCCv2. Starting from the MIC-OPCCv1 baseline, which performs octree-node occupancy estimation using depth-specific sub-modules, we incrementally activate each proposed design element from Section 4 and evaluate its impact relative to the G-PCCv14 anchor. Replacing node estimation with voxel estimation yields the first substantial improvement, increasing the BD-Rate gain from 20.2% to 21.8%, confirming that voxel-level supervision provides a more expressive learning signal for context modeling. Introducing the blender & distiller architecture further boosts performance to a gain of 25.4%, highlighting the effectiveness of multi-scale residual aggregation and voting-based distillation in stabilizing predictions across each hidden layer. Adding sequential grouping increases the gain to 28.1% but also raises decoding time from approximately ∼3 s to over ∼5 s per frame, illustrating the latency cost of strict autoregressive decoding. Replacing this scheme with the proposed progressive grouping strategy preserves comparable compression gains (28.6%) while avoiding additional runtime penalties by retaining partial parallelism. Finally, combining progressive grouping with the Sub-Module design yields the full MIC-OPCCv2 model, achieving the highest gain of 30.6% with a decoding time of approximately 5.1 s. This configuration represents the most favorable balance between compression efficiency and computational cost among all evaluated variants.

Overall, the ablation study demonstrates that each proposed component contributes meaningfully to performance improvements. The largest gains stem from voxel-based estimation, the blender & distiller architecture, and the progressive grouping strategy, whose combination enables MIC-OPCCv2 to substantially outperform the MIC-OPCCv1 baseline while maintaining practical decoding complexity.

## 8. Conclusions and Future Work

This paper presented MIC-OPCCv2, a neural entropy model for octree-based point cloud geometry compression that addresses the long-standing trade-off between R–D performance and decoding latency. By introducing multi-index convolution as a lightweight alternative to transformer-based attention and full 3D sparse convolution, the proposed framework enables explicit yet computationally efficient context modeling using only one-dimensional operations. Combined with the blender & distiller architecture and progressive grouping strategy, MIC-OPCCv2 achieves competitive compression efficiency while substantially reducing decoding complexity and memory overhead.

Experimental results demonstrate that MIC-OPCCv2 consistently outperforms traditional codecs such as G-PCC and achieves favorable R–D performance compared to recent neural approaches, including UniPCGC and ECM-OPCC, while offering up to a fourfold decoding speedup. The method generalizes effectively across heterogeneous data regimes, ranging from sparse LiDAR scans to dense surface reconstructions. At the same time, our results reveal a performance gap on highly dense and regular geometry when compared to fully autoregressive voxel-wise models such as SparseVoxelDNN. This gap can be attributed to the strict voxel-level dependency modeling employed by fully autoregressive methods, which is particularly effective for dense occupancy patterns but comes at the cost of substantially higher decoding latency and memory consumption. In contrast, MIC-OPCCv2 deliberately trades a small loss in compression efficiency for significantly improved runtime scalability, making it more suitable for latency-sensitive applications.

Further analysis indicates that the advantages of multi-index convolution are most pronounced in sparse-to-moderately dense point clouds, where explicit spatial indexing provides robust contextual cues while avoiding the non-local interactions and high memory footprint inherent in attention-based models. For highly regular dense surfaces, the fixed indexing scheme may introduce non-local voxel activations inside the perceptive field, which can limit achievable compression gains. Similarly, while progressive grouping—following principles previously explored in UniPCGC [9]—effectively balances contextual refinement and parallelism, more adaptive grouping schedules may further improve robustness across varying data distributions. These observations define clear applicability boundaries of the proposed approach and motivate future research directions.

Several directions remain open for future work. First, replacing fixed index permutations with learned or data-adaptive indexing strategies may improve spatial coherence and suppress non-local activations, particularly on dense and regular geometry. Second, more flexible grouping strategies, potentially driven by local occupancy statistics, could further optimize the R–D trade-off. Third, extending the framework to jointly compress geometry and attributes such as color, reflectance, or semantic labels would enable a unified neural point cloud codec. Finally, hardware-aware optimizations—including optimized gather/scatter primitives, quantization-aware training, and sparse acceleration—could further narrow the gap between theoretical and practical runtime performance.

In conclusion, MIC-OPCCv2 demonstrates that carefully designed index-based convolutional context models can achieve a favorable balance between compression efficiency, decoding speed, and scalability. Rather than maximizing compression performance at all costs, the proposed method explicitly targets practical deployment constraints, providing an effective and computationally tractable solution for next-generation point cloud transmission and storage.

## Figures and Tables

**Table 1:** A set of deep learning-based entropy models for geometric point cloud compression, grouped and categorized by their input features, primary context modeling components, output probability representations, and autoregressive level. The last two columns on the right summarize the relative compression performance gain^−1^ over G-PCCv14 and the decoding time for both sparse and dense point clouds.

Name	Input	Context Model	Output	Auto- Regressive	Sparse Dense	Time
MIC-OPCCv2	location, occupancy (binarized)	Index Convolution	voxels 0…1	semi	32.6% 30.6%	3.5 s 5 s
MSVoxelDNN	voxels	Masked 3D-Convolution			- −9.7%	- 58 s
VoxelDNN				fully	- 13.9%	- 640 s
NNOC					- 11.5%	- 1171 s
SparseVoxelDNN		Masked & Sparse 3D-Convolution			- 52.8%	- 229 s
SparsePCGC		Sparse 3D-Convolution		semi	22.0% 33.8%	1.2 s 1.9 s
UniPCGC					- 29.2%	- 0.57 s
RENO	occupancy (embedded)		octree nodes 1…255	none	17.8% -	0.1 s -
Voxel-Context Net	voxels	3D-Convolution			8.1% -	0.09 s -
MIC-OPCCv1	location, occupancy (binarized)	Index Convolution			19.6% 30.6%	2.5 s 5.0 s
OctSqueeze	location, occupancy, level, octant	Multi-Layer Perceptron (MLP)			−2.1% -	0.08 s -
OctAttention	occupancy, level, octant (embedded)	breadth-first transformer		fully	19.5% 9.7%	530 s 1229 s
ECM-OPCC				semi	32.6% 19.5%	- 19.8 s
EHEM					32.6% -	0.43 s -

**Table 2:** Comparison of R–D performance and decoding time on the SemanticKITTI dataset under different quantization schemes. For each baseline method, the original reported bpp and runtime are compared with the corresponding results obtained using our proposed framework under identical quantization settings. D1-PSNR is measured at L=12 and p=1.

	Quantization	D1-PSNR (dB)	bpp	Time (s)	Ours	
	*L* = 12	*p* = 1			bpp	Time (s)
ECM-OPCC	$\left\lfloor P\frac{2^{L}-1}{\max(P)-\min(P)}\right\rfloor$	77.2	3.39	-	3.41	4.0
EHEM	$\left\lfloor P\frac{2^{L}-1}{400}\right\rfloor$	60.0	2.6	0.43	1.99	3.0
RENO	$\left\lfloor P\frac{1000}{2^{18-L}}\right\rfloor$	61.8	2.9	0.05	2.41	3.6

**Table 3:** Average compression ratio (bpp) of 8iVFBv2, showing the gain over G-PCCv14 and coding time.

Point Clouds	GPCC	MIC-OPCCv2	UniPCGC	SparseVoxelDNN	ECM-OPCC
Redandblack	0.82	0.57	0.59	0.41	0.66
Loot	0.69	0.48	0.49	0.32	0.55
Thaidancer	0.70	0.50	0.51	0.33	0.58
Boxer	0.65	0.43	0.45	0.30	0.51
**Average Bpp**	0.72	0.50	0.51	0.34	0.58
**Gain^−1^**	0.0%	30.6%	29.2%	52.8%	19.4%
**Enc time (s)**	1.99	5.0	0.56	7.2	1.92
**Dec time (s)**	1.49	5.0	0.57	229	19.5
**Test Device**	i7	RTX3090	RTX4080	RTX3090	RTX3090

**Table 4:** Theoretical computational complexity of three leading point cloud compression architectures and their corresponding practical decoding times.

Method	Complexity	Example	Decoding Time (s)
Transformer	$O\left(n(3ckw+kw^{2})\right)$	ECM-OPCC	19.5
Minkowski Convolution	$O(m(ckw^{d}))$	UniPCGC	0.57
Multi-Index Convolution	$O\left(n(ckwi)\right)$	MIC-OPCCv2	5.0

**Table 5:** Ablation study of different autoregressive grouping strategies using the MVUB dataset [24]. Without Grouping: No voxel grouping is applied and decoding follows a fully parallel scheme. All voxel *v* of all octree-nodes $o_{t}$ are decoded a in single stage. Sequential Grouping: One voxel $v_{g}$ of all octree-nodes $o_{t}$ are decoded in parallel at each monotonic step g∈[1…8]. Progressive Grouping: The proposed strategy, which skips a subset of octree-nodes during early stages (g≤4) to reduce uncertainty and progressively decodes multiple voxel candidates per node in later stages (g>6) as contextual information becomes richer. (* Peak memory spikes observed during backpropagation.)

Method	Without Grouping	Sequential Grouping	Progressive Grouping
**Bits per Point**	1.25	0.76	0.64
**Estimation Accuracy**	84%	90%	92%
**Memory Peak per Point** *	14.6 KB	10.1 KB	12.9 KB
**Inference per Point**	1.076 ns	3.105 ns	2.594 ns

**Table 6:** Ablation study of the proposed model-splitting strategy, where dedicated sub-modules are assigned to different depth ranges of the octree. Table header labels denote model configurations; for example, 0–4–8–12 indicates three sub-modules split at octree layers 4, 8, and 12. To illustrate scaling behavior, each sub-module contains a number of convolution layers equal to its upper split boundary (i.e., 4, 8, and 12 layers). All configurations are evaluated on the SemanticKITTI dataset [19] for 10 epochs with 1000 samples per epoch. (* Peak memory spikes observed during backpropagation.)

Model Splitting	0–12	0–6–12	0–4–8–12	0–3–6–9–12	0–2–4–6–8–10–12
**Bits per Point**	4.169	3.883	3.722	3.720	3.715
**Estimation Accuracy**	84.2%	85.3%	85.9%	86.0%	86.0%
**Memory Peak per Point** *	116.9 KB	117.3 KB	117.1 KB	117.0 KB	117.2 KB
**Inference per Point**	0.532 ns	0.434 ns	0.405 ns	0.392 ns	0.367 ns

**Table 7:** Ablation study of the proposed methods from Section 4. All BD-Rates are relative to G-PCCv14. A checkmark (✓) indicates that the corresponding method is enabled in the evaluated configuration.

Method	MIC-OPCCv1					MIC-OPCCv2
**node estimation**	✓					
**voxel estimation**		✓	✓	✓	✓	✓
**Blender & Distiller**			✓	✓	✓	✓
**Sequential Grouping**				✓		
**Progressive Grouping**					✓	✓
**Sub-Modules**	✓					✓
**Gain^−1^**	20.2%	21.8%	25.4%	28.1%	28.6%	30.6%
**Decoding Time (s)**	2.2	2.8	2.9	5.4	5.3	5.1

## Data Availability

Source code and pre-trained models are published on https://github.com/bugerry87/mic-opcc (accessed on 12 February 2026).

## Abbreviations

BAC	Binary Arithmetic Coder
BD-Rate	Bjøntegaard Delta Rate
bpp	Bit Per (input) Points
ECM-OPCC	Efficient Context Model for Octree-based Point Cloud Compression
EHEM	Efficient Hierarchical Entropy Model for Learned Point Cloud Compression
FOC	Fast Octree Coding
G-PCC	Geometry-based Point Cloud Compression
MIC-OPCC	Multi-Index Convolution for Octree-based Point Cloud Compression
MLP	Multi-Layer Perceptron
MPEG	Moving Picture Experts Group
MVUB	Microsoft’s Voxelized Upper Bodies
NNOC	Neural Network Modeling of Probabilities for Coding the Octree Representation of Point Clouds
PCGC	Point Cloud Geometry Compression
PSNR	Peak Signal-to-Noise Ratio
R–D	Rate–Distortion
RENO	Real-Time Neural Compression for 3D LiDAR Point Clouds
RIDDLE	Lidar Data Compression with Range Image Deep Delta Encoding
UniPCGC	Towards Practical Point Cloud Geometry Compression via an Efficient Unified Approach
V-PCC	Video-based Point Cloud Compression
